# Neglected paths of transmission of milkborne brucellosis and tuberculosis in developing countries: novel control opportunities

**DOI:** 10.4178/epih.e2020073

**Published:** 2020-12-04

**Authors:** Arockiasamy Arun Prince Milton, Samir Das, Sandeep Ghatak

**Affiliations:** Division of Animal Health, ICAR Research Complex for NEH Region, Umiam, India

**Keywords:** Brucellosis, Education, Farms, Tuberculosis, Zoonoses, Transmission

## Abstract

Brucellosis and tuberculosis are lingering zoonotic infections that are endemic in many developing parts of the world, with considerable economic and health costs. Although guidelines for the control of these diseases exist, we highlight neglected transmission routes of these diseases. We show that informal, door-to-door marketing of unpasteurized milk provides an important route for disease transmission through kitchen cross-contamination. Furthermore, the practice of discarding the first strippings of milk at farms needs adjustment to avoid floor and environmental contamination. Herein, we propose handling guidelines and a design for a milk stripping collection vessel. We believe that taking action to block these hitherto unrecognized transmission routes will complement existing efforts and guidelines.

## INTRODUCTION

Brucellosis and tuberculosis are important chronic infections that are endemic in many parts of the world, especially in developing countries including India. These zoonotic diseases continue to inflict heavy burdens in terms of human morbidity and mortality worldwide and impose staggering economic costs [1,2]. Brucellosis, caused by various species of the genus *Brucella*, is a major milkborne zoonotic disease that principally originates in dairy animals, including cattle, sheep, and goats. Although estimates of the global costs of brucellosis are hard to come by, an available estimate from India indicates a median loss of US$3.4 billion annually with a prevalence up to 12% [2,3]. Tuberculosis, in contrast, is primarily caused by *Mycobacterium tuberculosis* and *M. bovis*, with evidence suggesting a substantial underestimation of the latter in causing human diseases. *M. bovis* causes extrapulmonary tuberculosis in humans and is predominantly a milkborne zoonosis originating from bovines [1]. Zoonotic tuberculosis is responsible for close to 10% of human tuberculosis cases in developing nations, and the production losses to the world cattle industry due to zoonotic tuberculosis amount to approximately US$3 billion annually [4]. With a lack of control programs and the considerable prevalence of zoonotic tuberculosis (approximately 7.3%), which affects a large number of cattle (approximately 21.8 million), large developing nations such as India pose particular challenges for global tuberculosis control efforts [4].

## THE CHALLENGES

Pasteurization of milk was traditionally the mainstay technique to stem the milkborne spread of brucellosis and tuberculosis. However, with rising trends in the consumption of minimally processed foods and unique marketing modes of milk in developing countries, including India, a large proportion of the population consumes unpasteurized milk. Both of these zoonotic diseases have been studied in detail over many decades, and multiple guidelines for the control of these diseases exist. However, we have observed certain unattended potential transmission routes that need to be urgently addressed.

**Figure 1.**
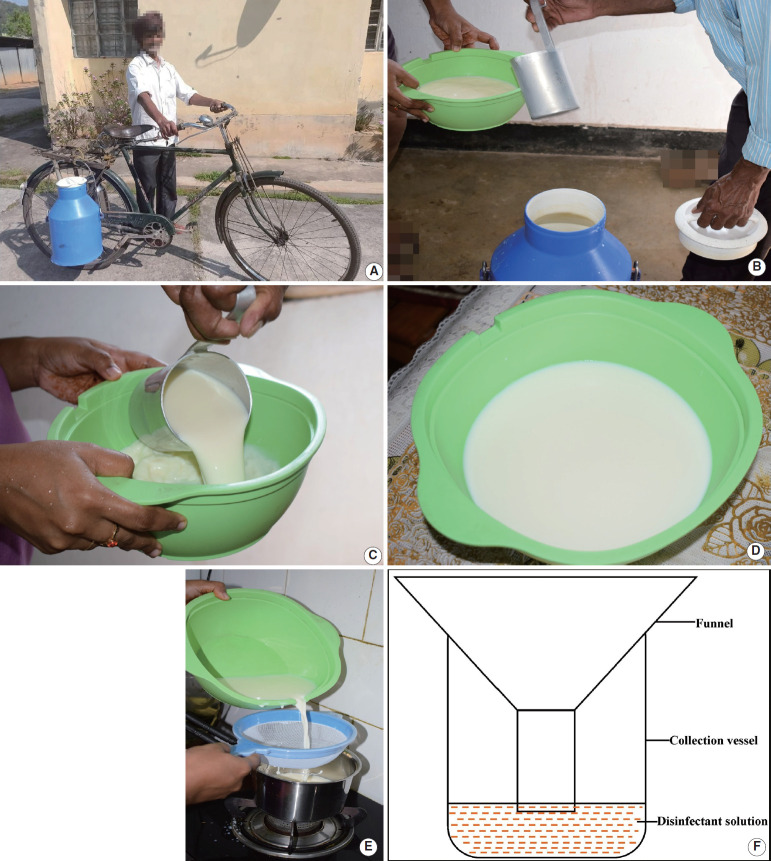
Informal retail milk handling by (A) door-step vendors, (B-E) consumers, and (F) proposed design of a safety container for collecting discarded first strippings at farms.

In developing countries such as India, primary milk production, distribution, and marketing are highly fragmented, and as noted by Kumar et al. [5], about two-thirds of the milk produced in India is actually marketed, of which 75-80% flows through informal, traditional, and unregulated channels. Our experiences as public health veterinarians demonstrated that milk is often produced at multitudes of small farms and small to medium-sized peri-urban dairies and is distributed unpasteurized to households in the vicinity of the farms through these local channels (Figure 1A). We further noticed that it is a common practice among customers to strain unpasteurized milk upon receipt to filter out visible dirt, followed by boiling the milk (usually for 5-10 minutes) before consumption (Figure 1B-E). While boiling effectively neutralizes/kills milkborne pathogens including *Brucella* and *Mycobacterium*, the strainer is often ignored and is just rinsed in water before being used for other kitchen applications. Moreover, it is common for customers to receive unpasteurized milk in one container (usually plastic or glass) and then change the container to boil the milk, while the first container is reused for other kitchen purposes following casual rinsing with water. We strongly suspect that these practices of straining milk and changing the containers used to handle raw unpasteurized milk create additional fomites that might potentially harbor milkborne pathogens, especially *Brucella* and *Mycobacterium*, as they are sturdy survivors in a moist environment. Therefore, these practices may lead to kitchen cross-contamination, posing a serious health threat to consumers.

In addition, at the farm level, we have observed another common practice that is also potentially hazardous, though farm hygiene is considered a major component for the control of brucellosis and tuberculosis. Due to the small-scale dairy production systems in India and other developing countries, machine milking is uncommon and milking is mostly carried out manually at farms. At dairy farms, it is common among milkers to discard the first few strippings of milk before the actual collection to avoid contamination of the entire collection. As the first strippings usually have a high bacterial count, we suspect that this practice significantly increases the floor contamination and may lead to transmission of brucellosis and tuberculosis to farm personnel and other susceptible animals, and may contaminate the farm environment. The environment may become contaminated with the discharges (excretions/secretions) of the infected animals, which can be picked up by other susceptible animal hosts. A number of reports have discussed the role of contamination of the immediate environment in the transmission of brucellosis and tuberculosis within dairy herds [6-8].

## THE SOLUTIONS

Intriguingly, we did not find any mention of these potential routes of disease transmission in commonly available guidelines for brucellosis and tuberculosis control. Considering the hazards, current practices, cultural preferences of consumers, and absence of appropriate guidance, we propose that retail consumers of raw and unpasteurized milk abstain from using a strainer upon receiving milk and boil the milk in the same container in which the milk was delivered. If the milk supplied is visibly polluted with dirt and straining is necessary, then the strainer should be boiled in water for at least 10 minutes and rinsed thereafter, before being put to other kitchen uses.

Regarding the practice of discarding the first strippings of milk, we propose that the first stripping should not be discarded on the floor; instead, it should be collected in a special vessel with a funnel placed inside (to avoid aerosol formation), containing a common disinfectant solution (e.g., 5% phenol, 2% glutaraldehyde, 0.5% sodium hypochlorite, etc.) to inactivate the pathogens (Figure 1F). The collected strippings may safely be disposed of later, following the established protocol of the farm.

## EDUCATION OF LIVESTOCK FARMERS AND CONSUMERS FOR CONTROL OF ZOONOTIC DISEASES

The control of zoonotic diseases relies on active participation by all stakeholders, including livestock farmers and consumers, along with a sustained and scientific approach to awareness-raising and education [9,10]. Multiple studies from around the world have highlighted the need for education and awareness-raising among farmers and consumers for the effective alleviation of zoonoses including brucellosis and bovine tuberculosis [11-17].

While combatting zoonoses through behavioral changes is very effective, achieving the desired behavioral modifications through education and awareness-raising is challenging. A number of available tools and methods for educating farmers may be utilized for such purposes [18]. Moreover, there is a need for improved risk communication and updated guidelines (incorporating the steps proposed herein) for early adoption by livestock farmers and consumers. Since optimal health education programs have a significant impact on the control of zoonotic diseases, customized training modules on kitchen hygiene and dairy farm hygiene focusing on the novel control opportunities proposed in this paper need to be developed and disseminated to curb the spread of zoonotic tuberculosis, brucellosis, and other similar diseases. Based on our experiences, we recommend the effective use of various digital tools (mass/social media) and creative communication channels (e.g., focus group discussions) to reach marginal livestock farmers, dairy cooperatives, and consumers around the world. Communication materials should be designed in local/vernacular languages with pictorial representations of these previously unrecognized transmission paths, as well as novel control methods, to maximize their impact among the native population. These steps need to be supported by advocacy programs for desirable behavioral modifications. Nevertheless, all these efforts need to be backed by strong political will and adequate funding mechanisms for them to be sustainable [9,10].

## CONCLUSION

It is an established fact that the control of any zoonotic infection in humans must start with preventing and controlling the disease in animal hosts, while also ensuring the safety of foods of animal origin. Considering our observations and potential solutions proposed, we believe that these simple and practicable measures may provide effective barriers against the spread of brucellosis, tuberculosis, and other milkborne zoonotic diseases through hitherto unheeded transmission routes. Raising awareness among consumers of unpasteurized milk and dairy farmers regarding these proposed interventions is therefore essential. Overlooking such unknown, yet critical transmission paths might prove costly for the control of these important zoonotic diseases, which already incur huge public health expenditures for their control worldwide.

### Ethics statement

Not applicable as the manuscript did not involve any experimentation.
